# Minimal difference between fractionated and single-fraction exposure in a murine model of radiation necrosis

**DOI:** 10.1186/s13014-019-1356-3

**Published:** 2019-08-13

**Authors:** Andrew J. Boria, Carlos J. Perez-Torres

**Affiliations:** 1 School of Health Sciences, Purdue University, 550 Stadium Mall Drive, Hampton Hall 1263A, West Lafayette, IN 47907 USA; 2 Purdue University Center for Cancer Research, Purdue University, West Lafayette, IN USA

**Keywords:** Fractionation, Mouse model, MRI, Radiation biology, Radiation necrosis

## Abstract

**Purpose:**

Despite the success of fractionation in clinical practice to spare healthy tissue, it remains common for mouse models used to study the efficacy of radiation therapy to use minimal or no fractionation. The goal of our study was to create a fractionated mouse model of radiation necrosis that we could compare to our single fraction model.

**Methods:**

Precision X-Ray’s X-Rad 320 cabinet irradiator was used to irradiate the cerebrum of mice with four different fractionation schemes, while a 7 T Bruker magnetic resonance imaging (MRI) scanner using T2 and post-contrast T1 imaging was used to track the development of radiation necrosis over the span of six weeks.

**Results:**

All four fractionation schemes with single fraction equivalent doses (SFED) less than 50 Gy for the commonly accepted alpha/beta ratio (α/β) value of 2–3 Gy produced radiation necrosis comparable to what would be achieved with single fraction doses of 80 and 90 Gy. This is surprising when previous work using single fractions of 50 Gy produced no visible radiation necrosis, with the results of this study showing fractionation not sparing brain tissue as much as expected.

**Conclusion:**

Further interpretation of these results must take into consideration other studies which have shown a lack of sparing when fractionation has been incorporated, as well as consider factors such as the use of large doses per fraction, the time between fractions, and the limitations of using a murine model to analyze the human condition.

## Introduction

Radiation therapy is essential to cancer treatment, with approximately 50% of cancer patients receiving radiation therapy and radiation contributing toward 40% of the curative treatments of the disease [1]. Fractionated radiation therapy is the most prominent technique for treating cancer with radiation [2] due primarily to fractionation allowing for the selective sparing of healthy tissue [1, 3]. Fractionation thus serves clinically to reduce the complications attributed to radiation therapy [4–7].

Though fractionation is the established approach clinically, it remains common in preclinical studies to perform experiments and generate animal models with high single fraction doses [8–12]. This unfractionated approach has practical advantages such as a shorter time commitment and avoiding potential confounds due to the potentially limited reproducibility of positioning for focal treatments. Even so, ideally animal models of radiation-induced injury should be performed with fractionated regimes to ensure that the radiation exposure is as human-like as possible.

We have recently published a mouse model of radiation necrosis generated with a large single fraction treatment [13]. Based on the logic above, our goal was to create a fractionated mouse model in order to treat mice in a way more similar to how humans are treated with radiation. Our hypothesis was that fractionation would provide a noticeable level of sparing to healthy tissue as seen in patients. However, the level of sparing was found to be minimal, with fractionation schemes predicted not to cause radiation necrosis based on our previous findings [13] instead causing radiation necrosis almost as severe as observed with single fraction doses of equal total dose.

## Materials and methods

All animal experiments were approved by the Purdue Animal Care and Use Committee. The general experimental framework included irradiation followed by MRI to track radiation necrosis lesion progression and finally post-mortem validation with histology.

### Setup and treatment

Irradiation was performed as previously described [13] so that our fractionated treatments are comparable to the single fraction data from that publication. Briefly, an X-Rad 320 (Precision X Ray, North Branford, CT) pre-clinical cabinet irradiator was used to deliver partial cerebrum doses to mice via a field 0.5 cm by 0.5 cm such that a single hemisphere was irradiated at a dose rate of about 2 Gy per minute. Female 8–9 week old BALB/c (Harlan, Indianapolis, IN) were irradiated once a day Monday through Friday as is usually done in the clinic.

### Fractionation

Four different radiation fractionation schemes were used: 5 fractions of 20 Gy, 10 fractions of 10 Gy, 5 fractions of 18 Gy, and 10 fractions of 9 Gy. We chose not to do more than 10 fractions over two weeks because in our previous work onset of pathology occurred at 2 or 3 weeks for 100 and 90 Gy in a single fraction respectively [13]. We were concerned that further protraction might lead to overlap of treatment and lesion onset. Based on the linear-quadratic model, the biologically effective dose (BED) and single fraction equivalent dose (SFED) were calculated for all four schemes and are included in Table 1 based off the commonly assumed alpha/beta ratio (α/β) for early and late responding tissue [14]. For both equations, n is the fraction number, d is the dose per fraction in Gy, and α/β in Gy is from the linear-quadratic model.

**Table 1:** BED and SFED calculated for three hypothetical α/β ratios for the four dose regimes used. The fraction number (n), dose per fraction (d), biologically effective dose (BED), and single fraction equivalent dose (SFED) are all included

Late Effects/Cerebrum α/β = 2 Gy	n	d (Gy)	BED (Gy)	SFED (Gy)
	5	20	1100	45.91
	10	10	600	33.66
	5	18	900	41.44
	10	9	495	30.48
Late Effects/Cerebrum α/β = 3 Gy	n	d (Gy)	BED (Gy)	SFED (Gy)
	5	20	766.7	46.48
	10	10	433.3	34.59
	5	18	630	42.00
	10	9	360	31.40
Early Effects/Tumor α/β = 10 Gy	n	d (Gy)	BED (Gy)	SFED (Gy)
	5	20	300	50
	10	10	200	40
	5	18	252	45.45
	10	9	171	36.65

The BED is calculated as given in Fowler [14] as

1 $$ BED=(nd)\times \left(1+\frac{d}{\alpha /\beta}\right). $$

The single fraction equivalent dose (SFED) is obtained from the BED by substituting d = SFED and *n* = 1, and then solving for SFED giving the following equation:

2 $$ SFED=\frac{-1+\sqrt{1^2+4\frac{BED}{\alpha /\beta }\ }}{\frac{2}{\alpha /\beta }}. $$

### Magnetic resonance imaging (MRI)

Inhaled isoflurane was used to anaesthetize mice prior to imaging. Mice received an intraperitoneal injection of 0.2 mL of Multihance (gadobenate dimeglumine; Bracco Diagnostics Inc., Princeton, NJ) prior to imaging diluted to a 1:10 ratio in saline. A Bruker BioSpec 70/30USR 7 T MRI (Billerica, MA) was used to image mice at multiple timepoints up to a final timepoint of six weeks. RARE T2-weighted images (Effective TE = 40 ms, TR = 4000 ms, Averages = 4) and MSME T1-weighted images (TE = 8 ms, TR = 500 ms, Averages = 4) were acquired. Twenty one slices with a 0.5 mm slice thickness were obtained for each scan type with the 3rd slice of both set of scans centered on where the olfactory bulbs and the rest of the cerebrum were separated. The matrix size of the scans was 128 pixels by 128 pixels with a field size of 15 by 15 mm^2^, with a corresponding resolution of ~ 0.117 mm.

### MRI data analysis

Radiation necrosis lesion quantification was assessed using a semi-automatic threshold segmentation algorithm as we have previously performed in this model [13]. Lesion is defined as regions of hyperintensity and hypointensity within the brain in this study with T1 and T2 images being analyzed independently. Both the upper and lower thresholds were chosen to be two standard deviations from the mean in normal mice. Brain segmentation and defining of lesion was carried out with a MATLAB (MathWorks, Natick, MA) program written in-house. Once the algorithm determined which voxels comprised the lesion, the lesion volumes is calculated by multiplying the total number of voxels by the unit volume for a voxel based on the scan geometry. In our scans, each voxel has a unit volume of roughly 0.007 mm^3^.

### Statistics

Quantitative data were compiled in Prism 8 (GraphPad Software, San Diego, CA) for the generation of plots and statistical analysis. When summary statistics are presented, data are shown as mean ± standard deviation. Two-Way ANOVA with a Tukey post-hoc test was used to compare the lesion volumes as a function of radiation scheme and time for T1 and T2 images independently.

### Histology

Mice were euthanized after final imaging with their brains collected and left fixed in 4% paraformaldehyde with graded alcohols being used for processing. Mouse brains were embedded in paraffin. Hematoxylin and eosin (H&E) staining was used on four micrometer sections of each mouse brain. An Evos XL (Life Technologies, Carlsbad, CA) digital inverted microscope was used to evaluate and photograph brain sections.

## Results

**Fig. 1:**
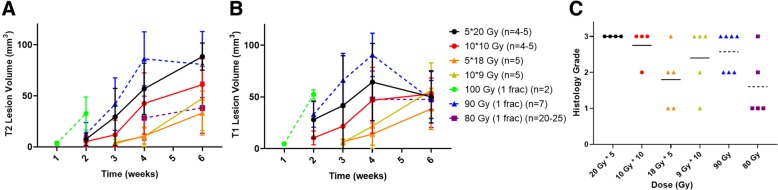
Lesion progression as a function of time post-irradiation. The figure shows the lesion size (in mm^3^) over time (in weeks) for mice that received multiple fractionation regiments as well as single fraction doses between 80 and 100 Gy for T2-Weighted (Panel **a**) and post-contrast T1-weighted (Panel **b**) MRI images as well as histological scores at 6 weeks post-irradiation (Panel **c**). The data is presented as mean ± standard deviation for lesion progression in Panels **a** and **b** with mean values present as bars in Panel **c**. The number of animals in each group (n) ranges from 2 to 25. Notice that the fractionated schemes’ lesion volumes are comparable to those of the three single fraction regiments against the expectations of the SFED seen in Table 1. Also, the post-radiation side effects for single fraction 100 Gy irradiations were severe enough that mice needed to be sacrificed at 2 weeks with data not available past this point. Histological scores for 100 Gy are not in Panel **c** since post-irradiation side effects required sacrificing these mice early at 2 weeks

The original purpose of our study was to create a fractionated mouse model of radiation necrosis that we could compare to our single fraction model. Mice were irradiated with four fractionation schemes and tracked up to 6 weeks with lesion volumes being measured. The lesion volumes measured were compared to what is generated with the same irradiation setup but single fraction doses [13]. Lesion development is observable on MRI in mice irradiated with all four fractionation schemes that is similar to what is observed in single fraction irradiations of 80 Gy or higher as is seen in Fig. 1 (Panels a and b). The two 100 Gy total fractionation schemes of 5 fractions of 20 Gy and 10 fractions of 10 Gy had lesion volumes most comparable to what is observed in single fraction irradiations of 90 Gy, while the two 90 Gy total fractionation schemes of 5 fractions of 18 Gy and 10 fractions of 9 Gy had lesion volumes most comparable to what is observed in single fraction irradiations of 80 Gy. Using a Two-Way ANOVA, there was no significant difference between 5 fractions of 20 Gy and 1 fraction of 90 Gy, 5 fractions of 18 Gy and 1 fraction of 80 Gy, and 10 fractions of 9 Gy and 1 fraction of 80 Gy at either 4 or 6 weeks post-irradiation on both T2 and T1. 10 fractions of 10 Gy and 1 fraction of 90 Gy showed a significant difference at 4 weeks post-irradiation on T2 (Tukey *P* = 0.0282) and T1 (Tukey *P* = 0.0492), but did not show a significant difference 6 weeks post-irradiation. Similarly, quantification of the radiation necrosis lesion on based on hematoxylin and eosin staining found no differences between the irradiation schemes at 6 weeks post-irradiation (Fig. 1 Panel c). A one-way ordinary ANOVA of the Histology Grade vs Dose (Gy) data found that the most significant Tukey post-hoc adjusted *p* value was 0.0617 between 20 Gy * 5 vs. 80 Gy, showing a lack of statistical significance in this data.

**Fig. 2:**
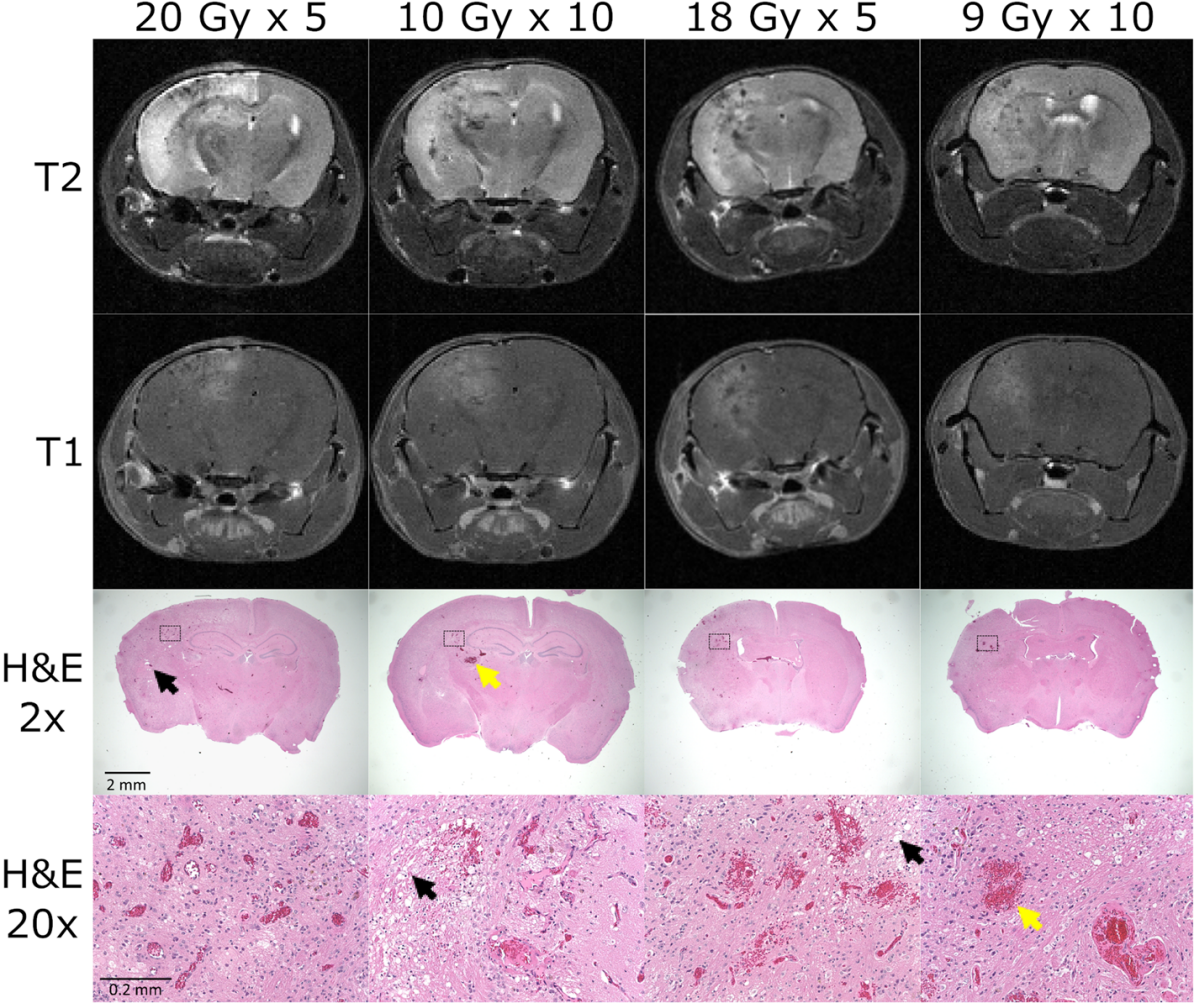
Representative MRI and histology images of murine radiation necrosis. T2-weighted (1st row), post-contrast T1-weighted (2nd row), and H&E images with a magnification of 2 and 20 respectively (3rd and 4th row) are presented for all four fractionation schemes: 5 fractions of 20 Gy, 10 fractions of 10 Gy, 5 fractions of 18 Gy, and 10 fractions of 9 Gy. The 3rd and 4th H&E images have black scales bars equal to 2 mm and 0.2 mm respectively. Areas of radiation injury on MRI (left hemisphere) correspond to visible pathology such as interstitial edema (black arrows) and hemorrhage (yellow arrows) on H&E images

Furthermore, brain swelling is observable in the former two fractionation schemes but mostly absent in the latter two fractionation schemes as observed in Fig. 2 with measurable damage caused by radiation on both MRI and Hematoxylin and eosin (H&E) being generally greater in the former than the latter. The results thus show that fractionation is less effective at controlling lesion development than expected with total dose being an important predictive factor of lesion development.

## Discussion

The purpose of this study was to create a fractionated mouse model of radiation necrosis that could be compared to our single-fraction model. All four fractionation schemes had single fraction equivalent doses less than 50 Gy. Though our single fraction irradiations of 50 Gy did not produce radiation necrosis within 26 weeks [13], all fractionated regimes led to measurable radiation necrosis on MRI and histology. The two 100 Gy total fractionation schemes had lesion volumes most similar to single fraction irradiations of 90 Gy, while the two 90 Gy total fractionation schemes were most similar to single fraction irradiations of 80 Gy.

The lack of sparing expected by fractionation observed is surprising, as this has been well evidenced in most tissues in laboratory animals leading to fractionation as the most common way we perform radiotherapy [15–18]. However, the sparing effects of radiation being less than anticipated in the rodent brain is not a new phenomenon. A prior report on a murine model of radiation necrosis [8] showed no difference when comparing a 60 Gy (50% isodose) treatment given in both 1 and 3 fractions. Similar results have been found when comparing fractionated to unfractionated regimes in a rat model of cognitive impairment [19] with single-fraction doses ranging from 11 to 17 Gy.

One potential explanation for the lack of sparing is the large fraction sizes and total dose in our study. However, various hypo-fractionated schemes are reported for late-responding tissues with similar fraction sizes to the low end of what we used which generally conform to the BED [7, 20, 21]. Another potential reason may be that we did not wait long enough in between delivering fractions. A prior report [22] gives a repair halftime for radiation necrosis to be 38.1 (6.9–76) hours based on human data. Thus, irradiating every other day instead of every day may result in additional sparing of damage.

An important limitation of our work is the rodent brain itself. The differences in levels of brain folding and white to grey matter composition between rodents and humans complicates the interpretations of any findings. Rodent models of radiation induced brain injury may not behave in a manner consistent with human disease. A clear example of this is the large doses that are needed to generate radiation necrosis in rodents [13]. We have also seen this in the past with models of radiation induced cognitive impairment not replicating the MRI deficits seen in humans [23]. Fractionation may not be as important a parameter in our murine model compared to parameters such as total dose, but we believe this is much more likely a feature of rodent brain irradiation models that is unlikely to be reflected in human patients.

## Data Availability

The datasets used and/or analysed during the current study are available from the corresponding author on reasonable request.

## Abbreviations

BED: Biologically effective dose
H&E: Hematoxylin and eosin
MRI: Magnetic resonance imaging
SFED: Single fraction equivalent dose
α/β: alpha/beta ratio
